# A Retrospective Study of Placenta Accreta, Percreta, and Increta in Peripartum Hysterectomies in a Tertiary Care Institute in Northeast India

**DOI:** 10.7759/cureus.11399

**Published:** 2020-11-09

**Authors:** Satya Dutta, Biswajit Dey, Sairem Chanu, Evarisalin Marbaniang, Nalini Sharma, Yookarin Khonglah, Vandana Raphael

**Affiliations:** 1 Pathology, North Eastern Indira Gandhi Regional Institute of Health and Medical Sciences (NEIGRIHMS), Shillong, IND; 2 Obstetrics and Gynaecology, North Eastern Indira Gandhi Regional Institute of Health and Medical Sciences (NEIGRIHMS), Shillong, IND

**Keywords:** pre-eclampsia, placenta accreta, caesarean section

## Abstract

Background: Abnormal placentation such as placenta accreta, increta, and percreta are frequent causes of post-partum hemorrhage, which results in maternal morbidity and mortality. A previous history of cesarean section, placenta previa, and pre-eclampsia are the important risk factors for abnormal placentation. A reliable antenatal diagnosis and planned surgical approach can reduce the incidence of maternal morbidity and mortality from massive hemorrhage.

Aim: To study the incidence of abnormal placentation and the association of various risk factors with abnormal placentation.

Material and methods: A retrospective study over a period of eight years in patients with peripartum hysterectomies due to abnormal placentation presenting with massive hemorrhage.

Results: We received a total of 10 emergency hysterectomy specimens during an eight‑year period. Of the cases, placenta accreta accounted for 40% (4/10), increta up to 40% (4/10), and percreta 20% (2/10). Analysis of these findings with parity showed 20% of the women were uniparous (2/10), and 80% were multiparous (8/10). Risk factor analysis showed previous cesarean sections in 40% (4/10), placenta previa in 50% (5/10), and pre‑eclampsia in 10% (1/10).

Conclusion: The present study highlights the incidence of abnormal placentation in a tertiary care institute in Northeast India. Placenta accreta and increta constituted the major forms of abnormal placentation. Multiparous women with placenta previa followed by previous lower segment cesarean section were more at risk of having abnormal placentation. These findings will guide in antenatal care by risk prioritization and management planning of these patients.

## Introduction

Abnormal placentation can be classified into three distinct entities such as placenta accreta, increta, and percreta [1]. In placenta accreta, the placental villi firmly adhere to the myometrium without an intervening decidual layer and in placenta increta, the trophoblastic layer extends deeply into the myometrium [1-2]. In placenta percreta, the villi penetrate through the myometrium reaching up to the uterine serosa, and may involve the adjacent structures [1-2]. All of these are important causes of post-partum morbidity and mortality [3]. Nowadays the incidence of abnormal placentation is increasing due to many factors like older maternal age at the time of delivery and proportionally more deliveries by cesarean sections [4-6]. Abnormal placentation also leads to late complications of pregnancy like pre-eclampsia, preterm labor, and postpartum hemorrhage [3]. The etiology of abnormal placentation is unknown; however, improper trophoblastic invasion and restricted endovascular invasion may be the pathological cause [7-8]. Here in this study, we tried to find out the incidence of abnormal placentation and various risk factors that increase the risk of abnormal placentation.

## Materials and methods

We present a retrospective cross-sectional study done on all the cases of abnormal placentation from the year January 2012 to December 2019.

All women with the antenatal diagnosis of abnormal placentation diagnosed by ultrasound or pregnant women with a history of placenta previa, multiple pregnancies, history of hypertension, previous history of cesarean section, advanced maternal age, women with a previous antenatal history of pre-eclampsia, and preterm labor were included in the study. Those with spontaneous separation of placenta intraoperatively or any other associated uterine pathology needing hysterectomy were excluded from the study. 

The clinical details and relevant obstetrics and gynecological history were noted from the histology requisition forms. Requisition forms with incomplete clinical details were also excluded from the study.

A total of 10 emergency peripartum hysterectomy specimens due to abnormal placentation were included in the study. We received peripartum subtotal or total hysterectomy specimens with or without in situ placentae. The site of abnormal placentation in the uterus and its distance from the cervix, the depth of the invasion into the myometrium, the involvement of any surrounding structures, and the presence of any laceration or perforation were noted during grossing of the specimens.

**Figure 1:**
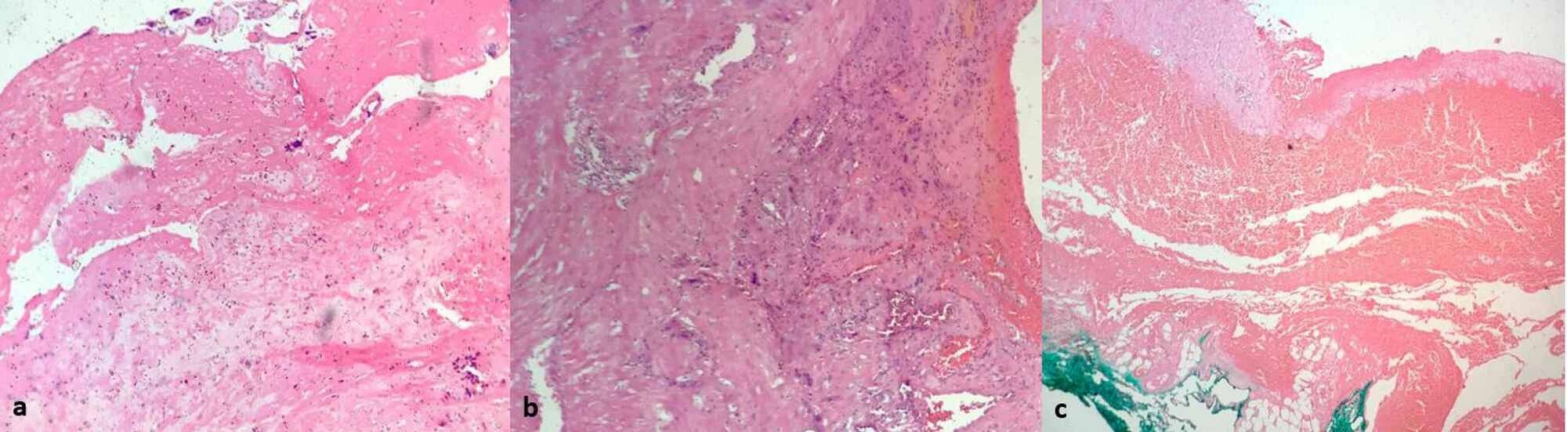
Histopathology of (a) placenta accreta showing placental villi adherent to myometrium (b) placenta increta showing trophoblastic cells extending into the myometrium, and (c) placenta percreta showing villi penetrating the whole thickness of myometrium (H & E, 100x).

Microscopic evaluation was performed on hematoxylin and eosin-stained sections. If there was no decidual layer between the placental villi and myometrium, a diagnosis of placenta accreta was made (Figure 1a). Deep myometrial invasion and myometrial perforation were seen in cases of placenta increta and percreta respectively (Figure 1b,c).

## Results

The age of the patients ranged from 22 to 38 years with a mean age of presentation of 30.7 years. A total of 10 hysterectomy specimens were received during the study periods. Four (40%) cases presented with antenatal bleeding, two (20%) cases presented with preterm labor, two (20%) cases presented with post-partum hemorrhage, and one case (10%) presented with uterine rupture at the time of labor.

Both placenta accreta and placenta increta constituted 40% of cases each. Placenta percreta was noted in the rest of 20% of cases (Table 1).

**Table 1:** Incidence of abnormal placentation in peripartum hysterectomies.

Abnormal placentation	Total no. of cases (n=10)	Percentage (%)
Placenta accreta	4	40
Placenta increta	4	40
Placenta percreta	2	20

Multiparity (80%) was more commonly associated with abnormal placentation (Table 2).

**Table 2:** Risk association of abnormal placentation with parity.

Parity	No. of cases (n=10)	Percentage (%)
Uniparous	2	20
Multiparous	8	80

Among various risk factors, placenta previa (50%) was the most commonly associated risk factors with abnormal placentation, followed by previous lower segment cesarean section (LSCS) (40%) and pre-eclampsia (10%) (Table 3). Among the four cases, which had a history of previous LSCS, three cases had more than three previous cesarean sections and one case had only one previous cesarean section.

**Table 3:** Association of risk factors with abnormal placentation. LSCS, lower segment cesarean section

Risk factors	No. of cases (n=10)	Percentage (%)
Previous LSCS	4	40
Placenta previa	5	50
Pre-eclampsia	1	10

## Discussion

The placenta accreta spectrum refers to a type of abnormal placentation that is abnormally adherent to the uterus. In placenta accreta, the placental villi invade the uterine myometrium without an intervening decidual layer and in placenta increta, the placental villi invade the myometrium. When the placental villi invade through the myometrium reaching up to the serosa and sometimes even perforate into adjacent structures like the bladder, it is called placenta percreta [1-2].

Abnormal placentation can be diagnosed accurately by microscopic examination assessing the degree of adhesion or invasion of the placental trophoblast layer into the myometrium [1-4]. In placenta accreta, there is the absence of Nitabuch’s layer or spongiosus layer of the decidua. Microscopically, the trophoblastic tissue invades the myometrium without intervening decidua [9]. This firm adherence of trophoblastic tissue to uterine myometrium leads to massive bleeding after delivery and the need for an emergency peripartum hysterectomy to prevent maternal mortality.

In the study by Breen et al., the incidence of abnormal placentation including placenta accreta, increta, and percreta ranged from 1 in 540 to 1 in 93,000 deliveries [10]. On the other hand, Fitzpatrick et al. demonstrated the rarity of placenta accreta/ increta/ percreta and estimated the incidence in the United Kingdom to be 1.7 per 10,000 maternities overall [11].

While the incidence of placenta accreta and increta in our study was comparable to that of Heena and Kumari, the incidence of percreta in our study (20%) was higher than that of Heena and Kumari (5.5%), which may be due to older maternal age among these women [12]. Increased maternal age is an independent risk factor for abnormal placentation [12-14]. In the present study, 80% of the women were multiparous, which corroborates the association of abnormal placentation with increasing parity [12]. 

Our study showed a higher association of risk factors with abnormal placentation i.e., women having placenta previa had a 50% risk of abnormal placentation and previous LSCS had a 40% risk. While Heena and Kumari showed women with previous cesarean section had a higher risk of abnormal placentation (55.5%) than placenta previa (33.3%). The risk of pre-eclampsia in our study (10%) was comparable to that of Heena and Kumari (11.1%) [12].

Placenta previa is a known risk factor of abnormal placentation along with the history of previous LSCS [15]. The risk association varies in different series. Wu et al. concluded that one or more prior cesarean deliveries, and placenta previa increase the incidence of placenta accreta [16]. The presence of placenta previa along with one prior cesarean delivery increases the risk of placenta accreta by 24% [17]. Whereas placenta previa in a patient with three or more prior cesarean sections increases the risk by 67% [17]. According to Finberg and Williams, around 75% of cases of placenta percreta are associated with placenta previa [18].

The study by Fitzpatrick et al. found that the risk of placenta accreta/ increta/ percreta increased in women with a previous cesarean delivery and placenta previa. But the authors did not find any linear correlation between placenta accreta/increta/percreta and the number of previous cesarean deliveries [11].

The major limitation of the study includes the retrospective nature of the study with data retrieved from the records available and thus limiting the sample size. Another limitation is the lack of data regarding the total number of deliveries during the study period.

## Conclusions

The present study highlights the incidence of abnormal placentation in a tertiary care institute in Northeast India. Placenta accreta and increta constituted the major forms of abnormal placentation. Multiparous women with placenta previa followed by previous LSCS were more at risk of having abnormal placentation. These findings will guide in antenatal care by prioritizing these patients based on risk factors and in planning their management.
